# Studies in Cancer Epigenetics through a Sex and Gendered Lens: A Comprehensive Scoping Review

**DOI:** 10.3390/cancers15174207

**Published:** 2023-08-22

**Authors:** Katherine Huerne, Sarah S. Jackson, Rina Lall, Nicole Palmour, Alison May Berner, Charles Dupras, Yann Joly

**Affiliations:** 1 Center of Genomics and Policy, Department of Human Genetics, McGill University, Montreal, QC H3A 0G1, Canada; 2 Division of Cancer Epidemiology and Genetics, National Cancer Institute, Rockville, MD 20850, USA; 3 Department of Epidemiology, Biostatistics, and Occupational Health, McGill University, Montreal, QC H3A 0G1, Canada; 4 Department of Genomics & Computational Biology, Barts Cancer Institute, Queen Mary University of London, London E1 4NS, UK; 5 Department of Social and Preventive Medicine, School of Public Health, University of Montreal, Montreal, QC H3T 1J4, Canada

**Keywords:** sex, gender, epigenetics, cancer, SAGER

## Abstract

**Simple Summary:**

This study aimed to assess how well sex and gender data was used in the field of cancer epigenetics. The researchers conducted a thorough review of 111 scientific studies focusing on colorectal, gastric, head and neck, hepatocellular carcinoma, and lung cancers. They found that only a small proportion (15.3%) explicitly analyzed sex and gender as a primary objective. While the majority (92.8%) provided some analysis of sex and gender as biological or social variables, only a few studies (6.3%) explicitly defined the terms “sex” and “gender”. Additionally, many studies (75.7%) incorrectly used these terms interchangeably, and there was inconsistency in their usage in 39.6% of the studies. In conclusion, the researchers emphasize the need for clear guidelines on incorporating sex and gender as variables in epigenetics research.

**Abstract:**

**Background**: Sex and gender are vitally important in the study of epigenetic mechanisms for various types of cancer. However, little has been done to assess the state of sex and gender-based analyses (SGBA) in this field. The aim was to undertake a critical evaluation of sex and gender representation, discussion, and data analysis within the cancer epigenetics field since 2010. **Methods**: A PRISMA-ScR scoping review was conducted with 111 peer-reviewed studies comprising of colorectal, gastric, head and neck, hepatocellular carcinoma, and lung cancers. Data extraction and a quality appraisal were performed by a team of epidemiologists and bioethicists. **Results**: Of the 111 included studies, only 17 studies (15.3%) explicitly stated sex and gender analysis to be their primary aim. A total of 103 studies (92.8%) provided a detailed analysis of sex/gender as a biological or social variable, while the remaining 8 studies (7.2%) only stratified results by sex/gender. Although sex and gender were a key facet in all the eligible studies, only 7 studies (6.3%) provided an explicit definition of the terms “sex” or “gender”, while the remaining 104 studies (93.7%) used the words “sex” or “gender” without providing a definition. A total of 84 studies (75.7%) conflated the concepts of “sex” and “gender”, while 44 studies (39.6%) were inconsistent with their usage of the “sex” and “gender” terms. **Conclusions**: Very few studies offered a robust analysis of sex/gender data according to SAGER guidelines. We call for clear and directed guidelines regarding the use of sex/gender as a variable in epigenetics research.

## 1. Introduction

### 1.1. Rationale

Sex and gender considerations are vitally important to cancer research, including its effect on cancer susceptibility, prognosis, and therapeutic response to cancer interventions [1]. In particular, epigenetics-based sex differences in cancer play a key role in gene regulation and expression, which can ultimately influence cancer outcomes [1]. These epigenetic sex differences may arise due a combination of inherited, behavioral, or environmental factors, the latter, which includes gender-based differences, therefore making both sex and gender key considerations in epigenetics-based cancer research. However, little has been done to assess the state of sex and gender-based analyses (SGBA) within the epigenetics field, such as evaluating the consistency in the usage of sex/gender terminology, or the appropriateness in statistical analyses when sex/gender is used as a biological or social variable. For example, correlational claims regarding epigenetic mechanisms driving sex differences in cancer research are often supported by statistically significant evidence. Yet, many of these frameworks of analyses fail to integrate sex or gender differences in a cognizant manner reflective of social or environmental differences found in the real world [2]. In studies involving sex/gender as a biological or social variable, as we will see, researchers often do not provide a working definition of sex/gender in their study or omit discussions on how sex/gender differences may impact clinical outcomes altogether.

As scientific findings shape and are shaped by existent discourse on sex and gender, it is important to be mindful of how researchers interpret and communicate sex/gender findings in a responsible, equitable, and representative manner. Epigenetics in particular, for its unique ability to integrate environmental effects as biological states, has the potential to affirm or dispel stigmatization of sex/gender-based differences in cancer expression. From an ethical perspective, ensuring both a cognizant representation and analysis of sex/gender differences is critical to shaping future discourse on the matter, particularly for historically underrepresented or misrepresented populations (e.g., women, intersex, transgender persons) in health research. Thus, true sex/gender equity in cancer epigenetics research goes beyond equal inclusion of males and females in a study, but also ensures that sex or gender data are accurately reported, analyzed, and discussed.

### 1.2. Objectives

We undertook a critical evaluation of the state of sex and gender discourse in peer-reviewed publications within the cancer epigenetics field since 2010, via a PRISMA-ScR scoping review. Working definitions of “sex” and “gender” were taken from internationally-recognized standards in health research (Box 1).

Box 1 **Key Definitions.**

Our sex/gender analysis is adapted from the World Health Organization [3], where:

*SEX* refers to the different biological and physiological characteristics of females, males, and intersex persons, such as chromosomes, hormones, and reproductive organs. Gender and sex are related to but different from gender identity.
*GENDER* refers to the characteristics of women, men, girls, and boys that are socially constructed. This includes norms, behaviors, and roles associated with being a woman, man, girl, or boy, as well as relationships with each other. As a social construct, gender varies from society to society and can change over time.

The state of sex or gender analysis was assessed using the Sex and Gender Equity in Research (SAGER) guidelines—the international standard for the reporting of sex and gender information in study design, data analyses, results, and interpretation of findings [4].

## 2. Methods

A scoping review was conducted to present the latest trends in sex/gender analysis in cancer epigenetics research (see the search summary and detailed methodology in Supplementary Materials S1 and S2). The protocol was reported following the PRISMA extension for scoping reviews (PRISMA-ScR) and made publicly available on Open Science Framework [5]. Peer-reviewed scientific literature was searched in PubMed, Google Scholar, and Scopus with keywords for cancer, epigenetics, sex, and gender. The eligibility criterion consisted of human studies published between January 2010 and August 2021 in English. Two independent reviewers performed the first- and second-round screening, data extraction, data analysis, and critical appraisal, with discrepancies mediated by consensus. From a total of 1411 initial hits, 390 duplicates were removed, 592 hits were excluded in the first round, and 243 studies were excluded in the second round. After classifying by cancer type, a total of 111 studies comprising the top 5 most populous cancer types (colorectal, gastric, head and neck, hepatocellular carcinoma, lung) returned from the search results of the epigenetics literature were ultimately included. Types of eligible studies included basic research studies, epidemiological studies, and reviews. Data extraction and a quality appraisal of all texts and figures was conducted by a team of epidemiologists and bioethicists to determine the state of SGBA according to SAGER and WHO guidelines. The results of the appraisal were used in conjunction with the results of the data extraction to provide a comprehensive report on the state of sex/gender findings in the cancer epigenetics field.

## 3. Results

### 3.1. Selection of Sources of Evidence

**Figure 1:**
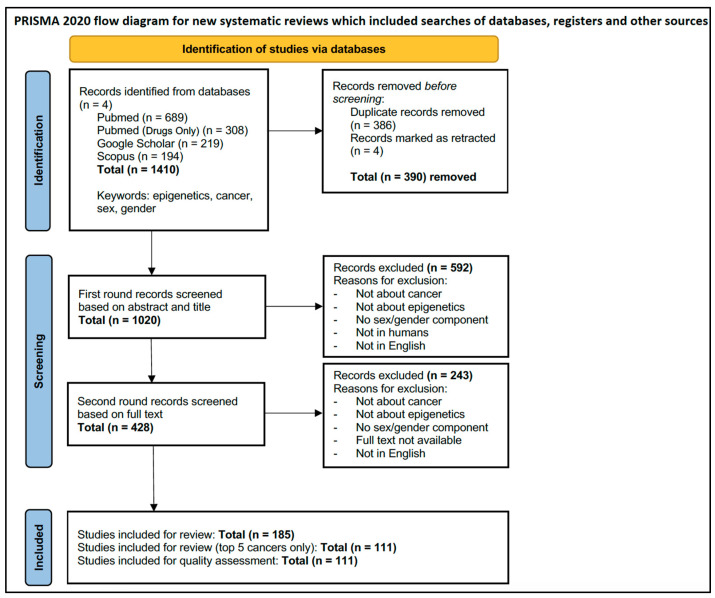
PRISMA-ScR Flow Diagram.

A flow diagram of the source selection and screening process is presented in Figure 1.

### 3.2. Characteristics of Sources of Evidence

**Figure 2:**
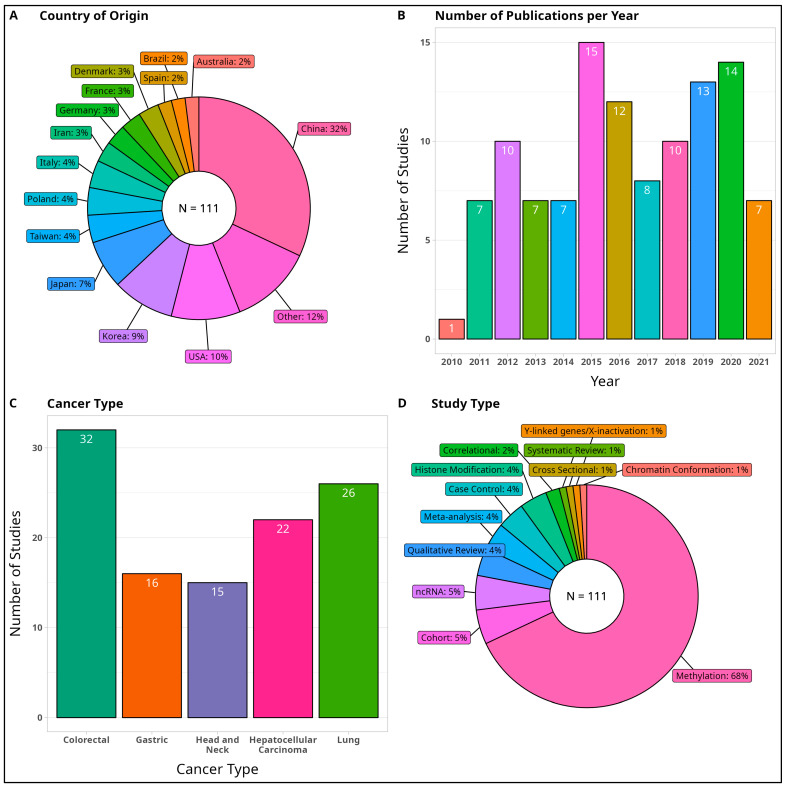
Characteristics of Sources of Evidence. Here, we describe the study characteristics of the total 111 eligible studies. (**A**) Country of Origin. (**B**) Number of Publications Per Year. (**C**) Cancer Type. (**D**) Study Type, where studies are broken down into basic research (methylation, histone modifications, chromatin conformation, non-coding RNA, drug studies, Y-linked genes), clinical research involving human participants (case control, cohort, correlational, cross sectional), and reviews (meta-analyses, systematic, non-systematic qualitative reviews).

**Figure 3:**
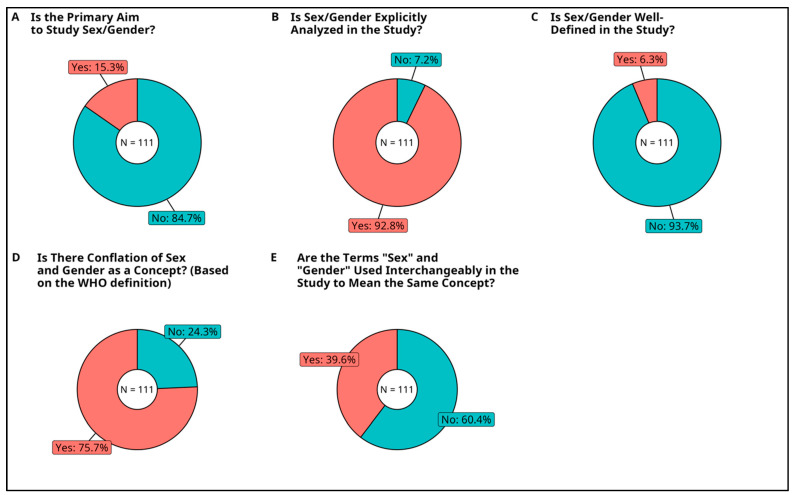
State of Sex and Gender Analysis in Selected Studies. In accordance with SAGER guidelines for sex and gender reporting and analysis in health research, we assessed the state of SGBA in the full text and Supplementary Materials of all 111 eligible studies, using key probing questions. (**A**) Is the Primary Aim to Study Sex/Gender? Yes = The main goal of the study was to examine sex/gender differences in cancer epigenetics and was explicitly stated in the aims statement. No = The aim of the study was about some other aspect of cancer epigenetics, and/or sex/gender was included without explicit emphasis. (**B**) Is Sex/Gender Explicitly Analyzed in the Study? Yes = A significant portion of the study utilized some qualitative or quantitative method of sex/gender analysis, with the methods of SGBA being well described. No = The study may have reported sex/gender characteristics but did not describe how sex/gender was analyzed in relation to the study’s aims. (**C**) Is Sex/Gender Well-Defined in the Study? Yes = A definition for sex or gender was provided explicitly in the text. No = A definition was not provided. (**D**) Is There Conflation of Sex and Gender as a Concept? (Based on the WHO definition) Yes = The context, discourse or cognizant understanding of the term sex or gender did not follow the respective definition provided by the WHO. No = The use of the term “sex” or “gender” followed the definition provided by the WHO. (**E**) Are the Terms “Sex” and “Gender” Used Interchangeably in the Study to Mean the Same Concept? Yes = The terms “sex” and “gender” were used interchangeably throughout the study to refer to the same concept. No = The terms sex and gender were used separately and consistently.

Here, we describe the study characteristics of the total 111 eligible studies (see Figure 1, Figure 2 and Figure 3).

#### 3.2.1. Country of Origin

The 111 studies originated from a total of 27 countries. The four most common countries conducting epigenetics cancer research include China (36 studies, 34.43%), USA (11 studies, 9.9%), Korea (10 studies, 9.0%), and Japan (8 studies, 7.2%). As most studies originated from East Asia, we recognize that the definition, conception, and use of the terms “sex” and “gender” may differ in these regions from the definitions provided by the WHO.

#### 3.2.2. Publication Year

From the trends shown in Figure 2, interest in sex and gender research within the epigenetics cancer field increased from 2015 onwards. The smaller number of publications seen in 2021 was because only 8 months’ worth of literature for that year was collected at the time of data extraction.

#### 3.2.3. Cancer Type

As this review was restricted to the top five cancer types, the number of studies for each cancer type is: colorectal cancer (32 studies, 28.8%), lung (26 studies, 23.4%), hepatocellular carcinoma (22 studies, 19.8%), gastric cancer (16 studies, 14.4%), and head and neck cancers (15 studies, 13.5%). When compared to overall epidemiological trends, the incidence and prevalence of these five cancers were all generally higher for men than women at older age, with notable sex differences in the specific expression or location of cancer subtypes [6,7,8,9,10].

#### 3.2.4. Study Type

Out of 111 total studies, the majority (88 studies, 79.3%) were concentrated on basic research, with 75 of the 88 basic research studies pertaining to studies on methylation alone. A breakdown of all study types is provided in Figure 2.

#### 3.2.5. Primary Aim to Study Sex and/or Gender

Although each study included a sex or gender component of some sort, considerations of sex or gender may not have been intentionally stated as the primary aim of each paper (see Table 1 for definition of this criterion). Based on the aims statement given by the 111 studies, only 17 studies (15.3%) described sex/gender as the primary aim, while 94 studies (84.7%) analyzed or discussed sex/gender as a secondary aim.

#### 3.2.6. Analysis of Sex or Gender

Although all studies involved SGBA in some way, we screened the methods section of each study to determine if sex/gender was thoroughly analyzed (either statistically analyzed or qualitatively discussed in detail), or if the results were simply stratified by binary sex (as defined in Table 1). Of the 111 studies, 103 studies (92.8%) performed a detailed analysis of sex/gender while 8 studies (7.2%) only stratified results by sex.

#### 3.2.7. Inclusion of Sex and Gender Definitions

As the words “sex” and “gender” may be variably used and conceptualized depending on national standards and cultural customs worldwide, it is important that any use of the terms sex and gender in research is cognizant of these contextual differences. After searching the abstract, full text, footnotes, appendix, supplemental material, tables, and figures of all 111 studies, only 7 studies (6.3%) provided an explicit well-defined and working definition of the terms “sex” or “gender”. Meanwhile, 104 studies (93.7%) used the words “sex” or “gender” in the study but did not provide a working definition. While it could be argued that basic scientists do not need to provide working definitions for universally recognized terms of “sex” and “gender”, our subsequent analysis on sex/gender usage suggests that these concepts are not as consistently represented as it would seem.

#### 3.2.8. Conflation of Sex and Gender as Concepts

Next, we sought to delineate if there were consistent conceptions of sex and gender across the epigenetics field as a whole. Although international guidelines such as the WHO have provided clear and distinct guidelines for the terms “sex” and “gender”, it was suspected that these conceptual frameworks may not be adopted in practical usage. We scanned all instances of the words “sex” and “gender” in each study to codify if their connotation was aligned with the WHO’s international guidelines. For example, a study was deemed to have conflated sex and gender if they used the word “gender” to refer to genetic or biological features that align with the WHO’s definition of sex. Of the 111 studies, 84 studies (75.7%) conflated sex and gender concepts together while 27 studies (24.3%) were clear in distinguishing sex from gender.

#### 3.2.9. Usage of Sex and Gender Terms Interchangeably

Another way of conflating sex and gender may be to use the terms interchangeably to mean either sex or gender throughout a single study. We scanned the full text of all 111 studies, and 44 studies (39.6%) used the terms interchangeably. On the other hand, 67 studies (60.4%) did not mix the two terms (e.g., authors used the word “sex” consistently and apart from the word “gender”, or vice versa).

#### 3.2.10. Inclusion of Non-Binary Sex/Gender Minorities

To examine if the field of cancer epigenetics engaged in considerations of sex and gender diversity, we assessed the abstract, full text, figures, tables, citations, appendix, and supplemental material for any mention, reference, or discussion of sex and gender beyond binary sex/gender categories (male/female; man/woman; XX/XY), such as inclusion of transgender, intersex, or two-spirit demographics. None of the 111 studies included mention of non-binary sex/gender minorities.

#### 3.2.11. Overall Sex Effect

A common objective in most studies was to detect statistically significant differences in epigenetic expression between the sexes. For example, a study detected an epigenetic effect in males if the epigenetic variable of interest reported a statistically significant finding for males in the results or conclusion of the study. If a multi-variable study found both a male and female effect, then both findings were independently recorded as an effect in males and an effect in females, separately. Of the 113 sex effects reported, 25 studies (22.1%) reported an epigenetic effect in males, 26 studies (23.0%) reported an effect in females, 47 studies (41.6%) found no differing effect between males and females, while 15 studies (13.3%) did not report any significant sex effect.

#### 3.2.12. Gender-Responsive Assessment Scale

**Figure 4:**
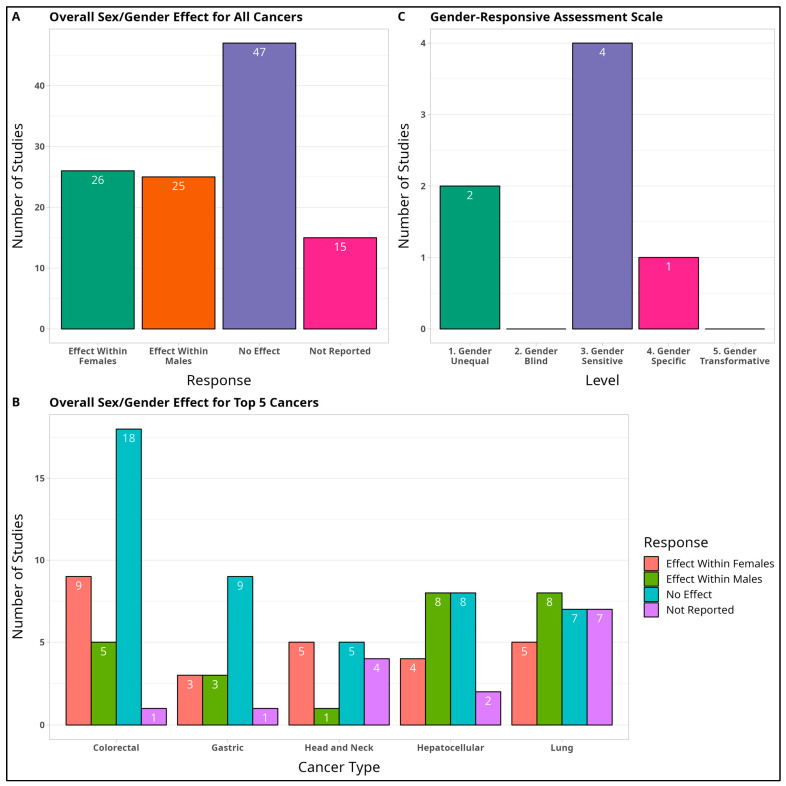
Summary of Sex and Gender Effects. A summary of sex and gender trends as reported by the authors of the total 111 eligible studies. (**A**) Overall Sex/Gender Effect for All Cancers and (**B**) Overall Sex/Gender Effect for Top Five Cancers. Effect Within Males = The study detected a significant positive or negative trend within males for the epigenetic variable of interest. Effect Within Females = The study detected a significant positive or negative trend within females for the epigenetic variable of interest. No Effect = The study did not find any significant positive or negative sex or gender-based trend for either males or females. Not Reported = The study did not report a sex/gender trend for the variable of interest. (**C**) Gender-Responsive Assessment Scale. For the seven studies which provided a gender analysis component, an assessment of their analysis was scored according to WHO guidelines [6]. Gender Unequal = The study discussed gender in a way that perpetuates gender inequality by privileging men over women, or vice versa. Gender Blind = The study discussed gender in a way that ignores gender norms, roles, and relations based on the principle of being fair to everyone. Gender Sensitive = The study discussed gender in a way that considers gender roles, relations, and norms, but provides no remedial action. Gender Specific = The study discussed gender in a way that considers gender roles, relations, and norms, by targeting men or women explicitly. Gender Transformative = The study discussed gender in a way that addresses the causes of gender-based health inequities and includes ways to transform harmful gender norms, relations, and roles.

To assess the quality of gender discussion and analysis in studies that mentioned gender, we performed a qualitative assessment according to the WHO’s Gender-Responsive Assessment Scale (2010) [11]. This tool was originally developed to evaluate the gender responsiveness of specific programs and services for women and other gender minorities. The assessment criterion (see Table 1 for description) was repurposed to evaluate how studies responded to gender-related issues in the design, implementation, and evaluation of their research aims. Out of 111 studies, only 7 studies (6.3%) mentioned gender, of which 2 studies were ranked gender unequal, 4 studies were ranked gender sensitive, and 1 study was ranked gender specific. Notably, no studies were ranked gender transformative, which is the highest standard for gender-sensitive reporting. See Figure 4’s legend for definitions of the different rankings.

### 3.3. Critical Appraisal within Sources of Evidence

A critical appraisal of the state of sex and gender-based analysis was performed according to a self-adapted protocol from the SAGER guidelines (see protocol in Supplementary Materials S3; results in Supplementary Materials S4). Sex as a biological variable was relevant in all the 102 basic research articles examined in the appraisal (review articles and one male-only basic research study were excluded for the critical appraisal). While all articles used the concepts of sex and gender, only one article defined these concepts explicitly. Further, 58 studies (56.7%) used the term “gender” when they meant “sex”, and 8 studies (7.8%) used the terms “sex” and “gender” interchangeably, as defined by SAGER guidelines. Thirty-five studies (34.3%) used the term “sex” appropriately. Nine studies (8.8%—four liver, three CRC, two lung, and none of the gastric or head and neck papers) made it clear what aspects of sex they were referring to. The other 94 studies (92.2%) did not specify which aspects of sex (e.g., chromosomes, sex hormones, etc.) or gender (e.g., expression, social roles) they were studying. Given extant literature, all the studies should have considered sex in their analysis, though only fifteen articles (14.7%) prespecified sex-based analyses (five CRC, five HCC, three gastric, one lung, and one head and neck) and only nine of those articles (8.8%) made references to sex in the research questions or hypotheses. Nine of the articles (8.8%) cited prior studies that support the existence of sex differences in the background and nine articles (8.8%) also detailed the extent to which past research has taken sex into account.

For the critical appraisal, only studies that included both males and females within the same study were included (single-sex studies of only males or females could not be disaggregated by sex). In this regard, the population was appropriate to capture gender and/or sex-based factors as all the studies could have disaggregated their results by sex. Despite this, we were unable to evaluate whether the inclusion and exclusion criteria were well justified with respect to sex and/or gender, as only four articles (3.9%) provided details on this in their inclusion/exclusion criteria. Similarly, the data collection method proposed for each study may not have been appropriate for investigation of sex and/or gender as the authors conflated these concepts (thus it was unclear what aspect of sex was being studied).

There were only 16 studies (15.7%) in which the analytic approach was appropriate and rigorous (e.g., looked at the exposure and outcome relationship separately by sex) enough to capture sex-based factors. One study did not perform any analyses of sex. Sex was used as an adjustment variable in only four (3.9%) of the studies. For most studies (91 studies, 89.2%), the authors presented univariate associations between sex and methylation. These analyses were unadjusted for factors that may influence methylation and differ between the sexes, like age. Fourteen studies (13.7%) disaggregated their results by sex. These studies examined associations between methylation and the main outcome (e.g., cancer incidence and/or survival) separately by sex. Of these fourteen studies, three were about CRC, eight from liver cancer, two from head and neck, and one from lung cancer. However, the remaining 88 studies (86.3%) did not disaggregate their results by sex, so any sex differences in the effect of DNA methylation and cancer could not be elucidated.

Twenty-three studies (22.5%) discussed sex-based analysis in the discussion section. Seventy-nine studies (77.5%) did not mention sex in the discussion and none of them noted that the lack of disaggregation of their results by sex was a limitation. Promising findings for one sex could be lost due to failure to disaggregate results. Only 12 studies (11.8%) included relevant ethical issues that might have particular significance with respect to sex in their study design.

### 3.4. Results of Individual Sources of Evidence

Here, we report the main findings from select high-quality studies within the top five cancer types and highlight how these high-quality studies can still hold limitations or inconsistencies within its sex/gender analysis component. High quality studies are defined as studies with high sample sizes (*n* > 100), use of a control group, includes statistically significant findings whether positive or negative (*p* ≤ 0.05 where possible), and present no serious limitations in overall study design (apart from the sex/gender analysis) as outlined in the critical appraisal above. A summary of the main findings is presented in Table 1.

#### 3.4.1. Colorectal Cancer (CRC)

Out of the 32 colorectal cancer studies, the majority (18 studies, 56.3%) did not report significant sex or gender-based epigenetic differences. Although this may imply that most epigenetic mechanisms for colorectal cancer are not influenced by sex/gender, the three reviews about this cancer type reported distinct sex/gender effects in females. A systematic review conducted by Jia et al. suggests that CpG island high methylation phenotype (CIMP-high) tumors are more often present in women than in men [12]. Another review by Kim et al. examining sex and gender differences in CRC reported that women are more prone to right-sided colon cancer than men, which is the more aggressive form of neoplasma [13]. The study argues that, in addition to the sex-based genetic/epigenetic factors or dietary habits, socio-cultural factors such as barriers to screening and diagnosis for women can explain gender-specific differences in CRC risk, which is also a widely recognized difference within clinical practice. Interestingly, a meta-analysis of 24 articles and 2025 total CRC patients by Liang et al. found no statistically significant association between APC methylation status and sex (M vs. F: OR 1.55, 95% CI 0.88–1.52, *p* = 0.31, I^2^ = 0%) in the early diagnosis of colorectal cancer, although this is just one of many epigenetic events that may drive tumorigenesis [14].

A total of nine studies (28.1%) reported a sex-based effect in females. Notably, an integrative genome-wide DNA methylation analysis performed by Fennell et al. reported that CIMP-H1 subgroup was enriched for female patients (18 of 23, 78.3%; *p* < 0.001) for a population of 216 unselected colorectal cancers (colorectal cancer *n* = 216 with matched normal *n* = 32 samples) [15]. However, only a univariate analysis for sex was performed without adjusting for other clinicopathological factors such as age or cancer stage. Another methylation study from Bi et al. using 432 primary CRC patients and 434 cancer-free controls reported that CHST7 methylation in white blood cells is positively associated with CRC risk, especially in females (OR = 7.704, 95% CI 4.222–14.058; *p* < 0.001) versus males (OR = 1.810, 95% CI 0.782–4.187; *p* = 0.157), and may potentially serve as a blood-based predictive biomarker for CRC risk [16].

Meanwhile, five studies (15.6%) reported a sex-based effect in males, but generally the studies were weaker with smaller sample sizes (*n* < 100) and inconsistent use of non-cancerous controls. Notably, a microRNA hypermethylation study by Kashani et al. with 51 polyps, 8 tumors, and 14 normal colon mucosa controls revealed that miRNA-137 hypermethylation was significantly higher in male patients (*p* = 0.002), although only a univariate analysis was performed without adjustment for important cofounders [17]. Another study by Rawłuszko et al. involving 52 CRC patients with paired normal tissue controls detected significantly lower amounts of 17β-Hydroxysteroid dehydrogenase type 1 (HSD17B1) gene transcript levels in male cancerous tissues compared to controls (transcript *p* = 0.0388; protein *p* = 0.2832), with no differences in gene versus protein levels in females (transcript *p* = 0.2340; protein *p* = 0.2119) [18]. Likewise, only a univariate analysis was performed without adjusting for co-variables.

#### 3.4.2. Gastric Cancer (GC)

Of the 16 studies, half (8 studies) were conducted in China, and a majority (10 studies, 62.5%) either did not report or reported no significant sex/gender differences. Notably, a meta-analysis conducted by Hu et al. of MLH1 promoter methylation levels using 4654 GC patients and 3669 non-cancerous controls revealed that methylation levels did not correlate with sex (OR = 0.73, 95% CI = 0.51–1.06, *p* = 0.097) [19]. From existing publications, it is difficult to determine if GC has a true sex effect, as most other studies which reported this outcome used small sample sizes, were not properly controlled, or did not compare differences between male and female populations.

A total of two studies (12.5%) reported an epigenetic effect in females. Notably, a study by Ghadami et al. which used 53 tumor tissues and their non-neoplastic control counterparts to study the CpG island methylation expression level of lysine 63 deubiquitinase (CYLD) gene promoter in gastric adenocarcinoma revealed that the expression level of CYLD mRNA was significantly decreased in females [Male: 95% CI −0.1451 to 0.2651, *p* = 0.5622; Female: 95% CI 0.2709 to 0.8891, *p* = 0.001] [20]. However, this study did not control for covariates and included a disproportionately higher number of females. Given the limitations in study design, it cannot be confirmed whether a true sex effect exists for females. One further study by Qu et al. on differential microRNA expression profiles using 386 gastric adenocarcinoma samples from The Cancer Genome Atlas (TCGA) database also revealed a microsatellite instability (MSI) association with sex, with female GC patients (25.2%) more likely to develop MSI-H type tumors than male GC patients (13.3%, *p* = 0.014) [21]. However, no controls were used, and the number of male participants was roughly double the number of female participants. Though MSI is not an epigenetic effect itself, the study presents evidence that microRNAs regulate MSI status, hence its relevance. However, authors do not postulate a mechanism by which differences in microRNA regulation might account for the sex effect observed.

There were also three studies (18.75%) which reported an epigenetic effect in males. Notably, a study by Waraya et al., which examined DNA methylation aberrations along key p53 pathway genes (PGP9.5, NMDAR2B, CCNA1 and p53) in 163 primary gastric cancer samples, with higher methylation aberrations reported in males (Mann−Whitney *p* = 0.003; log rank RFS: *p* = 0.03, OS: *p* = 0.1) [22]. However, controls were not used for the sex/gender-based analysis portion of their study, and they did not control for covariates. Another study by Uesugi et al., which examined high methylation epigenotype (HME) in minute intramucosal neoplasia (MIMN), found a sex distribution difference between MIMN and non-MIMN, where the relative ratio of men to women was significantly higher in MIMN than in non-MIMN cancers (men/women in MIMN > men/women in non-MIMN; *p* = 0.0074) [23]. However, like many other studies, covariates were not accounted for in their sex analysis and as such it likewise cannot be confirmed whether a sex effect does indeed exist for females.

#### 3.4.3. Head and Neck Cancers (HNC)

Of the fifteen studies, the majority (nine studies, 60%) did not find a sex/gender effect (five studies reported no effect, four studies did not report any effect). Most studies which reported no sex/gender effect had less than 100 total participants or did not control for the sex/gender analysis. Exceptionally, a study by Shen at al. examining DNA-methylation-based signatures for oral squamous cell carcinoma (OSCC) prognosis used 313 OSCC cases downloaded from the TCGA [24]. Using univariate linear regression adjusted for sex (M/F) and overall survival, then multiple linear regression adjusted for sex (M/F), high risk score, age, HPV status, stage, smoking status, and tumor grade, it was determined that sex was not significant to survival (univariate: HR 1.03 [0.69–1.53], *p* = 0.874; multivariate: HR 1.05 [0.65–1.70], *p* = 0.821).

Interestingly, almost all remaining studies save one (five studies total, 33.3%) reported an epigenetic effect in females. A study by Misawa et al. evaluated the prognostic value in promoter methylation status of galanin (GAL) and galanin receptor 1/2 (GALR1/2) in HNCs [25]. Using a total of 202 patients with head and neck squamous cell carcinoma (43 hypopharynx, 42 larynx, 59 oral cavity, and 58 oropharynx tumor samples), it was found that methylation was positively correlated with female sex (*p* = 0.008). Interestingly, methylation was higher in males (0.98 ± 0.77) than females (0.29 ± 0.49; *p* = 0.030) for hypopharyngeal cancers, while methylation was higher in females (1.75 ± 1.06) than in males (0.89 ± 0.94; *p* = 0.001) in oral cancers. Another study by Langevin et al. on microRNA-137 promoter methylation from 99 squamous cell carcinoma of head and neck (SCCHN) patients and 99 cancer-free controls reported that methylation levels were associated with female sex, with female cancer patients having five times the odds of having microRNA-137 promoter methylation than males (OR = 5.30, 95% CI 1.20–23.44) [26]. The sex association was derived via a multivariate logistic regression adjusted for sex, BMI, dentures, alcohol use, and total years smoking.

Finally, the one study by Challouf et al. which reported a sex effect in males examined DNA hypermethylation of nasopharyngeal carcinoma in 36 cancerous and 19 non-tumor nasopharyngeal tissues [27]. Of the 10 genes (RASSF1A, SHP1, DAPK, P16, RARβ2, GSTP1, TIMP3, APC, CDH1, or MGMT) examined, a positive association was found between SHP1 and males (*p* = 0.07). However, sample sizes were small and only a univariate analysis was performed (the study did not adjust for covariates). It is interesting to note that the prevalence of head and neck cancers in the real world primarily affect males, while overall, the included studies overwhelmingly detected an epigenetic effect in females, usually hypermethylation. However, most studies lacked large sample sizes with robust sex/gender analysis and thus the true effect remains inconclusive. Additionally, there was a notable absence of discussions about possible reasons for the observed gender or sex effects in review articles for this cancer type.

#### 3.4.4. Hepatocellular Carcinoma (HCC)

Of the 22 hepatocellular carcinoma (HCC) studies, more than half (14 studies, 63.6%) were conducted in China, with an additional 4 studies (18.2%) arising from other East Asian countries. A total of eight studies (36.4%) reported no significant sex or gender-based epigenetic differences, while two studies (9.1%) did not report any specific sex effect. Notably, a systematic review and meta-analysis by Lu et al. on runt-related transcription factor 3 (RUNX3) methylation and hepatocellular carcinoma association in 491 HCC patients and 409 tumor-free controls (from 7 studies) found no sex association (OR: 1.30, 95% CI = 0.48–3.50) [28]. Another study by Zhang et al. examining the correlation between P14ARF gene DNA methylation and primary liver cancer in 87 cancerous liver samples and adjacent tissue controls found no difference with respect to sex (*p* = 0.11) [29].

Of the remaining studies, eight studies (36.4%) reported an epigenetic effect (of typically hypermethylation or positive correlations of epigenetic markers) in males, and four studies (18.2%) reported an epigenetic effect in females, which corresponds with trends that HCCs tend to be more prevalent in males. Notably, a computational framework of methylation signature analysis by Meunier et al. using 738 CHC samples and adjacent normal controls from two liver cancer cohorts (Liver Cancer France and The Cancer Genome Atlas Liver Hepatocellular Carcinoma) revealed a positive and strong sex association of CpG island methylation to X chromosome inactivation in female populations (*p* = 5.0 × 10^−64^), with 96% of regions studies located within active transcriptional start sites of X chromosome genes [30]. Another study by Zhou et al. explored the diagnostic potential of cyclin-dependent kinase-like 2 (CDKL2) methylation in HCC patients [31]. Using 178 HC tissues, 169 adjacent non-tumor tissues and 24 normal liver tissues, it was revealed that CDKL2 methylation was associated with sex (*p* = 0.023), with methylation levels of CDKL2 appearing much higher in female patients (*p* = 0.037).

In males, a study by Nishida et al. which aimed to identify methylation-silenced tumor suppressor genes in early hepatocarcinogenesis as biomarkers, with a sample of 482 liver tissues and adjacent normal controls, found that males were significantly associated with shorter time-to-HCC occurrences in the univariate model (HR = 2.03, 95% CI 1.06—4.08, *p* = 0.0324) but not in the multivariable model (HR: 2.15, 95% CI 0.93, 5.27, *p* = 0.0742) [32]. Another study by Wang et al. examined the association of CpG sites in gene TNFRSF12A to HCC patients using a cohort of 345 TCGA HCC patients and paired normal tissue [33]. A Kaplan–Meier survival analysis showed that male HCC patients with hypomethylation in cg26808293 exhibited poorer prognosis than hypermethylation patients (*p* = 0.004). However, as the cohort specifically included those with alcohol abuse history, the sex differences were not observed in male HCC patients with non-alcoholic risk factors. In parallel, the authors acknowledged a gender behavior component where males were more likely to consume alcoholic beverages than females. A strong risk factor for HCC is alcohol use, and it remains to be discerned at the epigenetic level whether these observed “sex” differences are due to sex biology or gendered social differences.

Finally, a review by Ye et al. sought to delineate sex and race-based trends in hepatocellular carcinoma much like our current review [34]. They noted the real-world prevalence was more common in men than women, alongside some sex-specific effects such as a genetic variant rs11453459 in the protein phosphatase 2 scaffold subunit α gene (PPP2R1A) promoter region having a more protective effect in women than men, or more LINE1 hypomethylation levels in male than female HC patients. Nonetheless, the authors similarly concluded there was insufficient evidence indicating that it is the epigenetic component which leads to the observed sex differences in HCC incidence. The same limitations were cited, such as imbalanced sample sizes and lack of appropriate covariate control.

#### 3.4.5. Lung Cancer

Out of the 26 studies, 7 studies (26.9%) reported no significant sex related effect, while 7 studies (26.9%) did not report any sex/gender effects altogether. Notably, a study by Li et al. examining the association of RAR-β gene methylation in 167 non-small cell lung cancer (NSCLC) patients and 105 controls revealed no relationship between gene methylation to sex (Chi-square *p* = 0.052; logistic regression, *p* = 0.074, OR = 0.46, 95% CI = 0.193–1.094) [35]. However, the study was a univariate analysis with no control for other confounders. A similar study by Hwang et al. examined the association of heparan sulfate (glucosamine) 3-O-sulfotransferase 2 (HS3ST2) hypermethylation in 324 NSCLC patients and revealed no association by sex (*p* = 0.33) [36]. However, the study sample consisted of a higher proportion of males and did not adjust for confounders.

Comparable to real-world prevalence, a slight majority of remaining studies (eight studies, 30.5%) reported an epigenetic-based effect in males while five studies (19.2%) reported an effect in females. For studies that reported an effect in females, none of the studies included a control population. Two studies examined the association of epidermal growth factor receptor (EGFR) mutations in lung cancer patients. A cohort study by Peddireddy with 126 never smokers with NSCLC reported that activating EGFR mutations was significantly higher in females (67.6% Female vs. 26.7% Male; *p* = 0.002) after a multivariate logistic regression model controlling for sex, environmental smoke, age, and history of cancer (OR 5.13, 95% CI 1.47–18.0, *p* = 0.0105 for females) [37]. A study by Nguyen et al. using 139 lung adenocarcinoma also reported that EGFR mutations were more common in females (*p* < 0.001), but there was no sex difference in EGFR methylation (*p* = 0.893) or EGFR overexpression [38]. However, only a univariate analysis was performed.

Notably, for studies which reported an effect in males, the majority (six out of nine studies) were completed in China or Taiwan, where a large proportion of smokers are male [39,40]. A study by Lu et al. examined the correlation between orphan nuclear receptor NR0B1 activation and DNA methylation in 160 adenocarcinoma cells and paired adjacent noncancerous tissues [41]. It was concluded that CpG sites on the NR0B1 promoter were almost unmethylated in males, whereas fewer than 50% were unmethylated in females (*p* = 0.0124), with NR0B1 activation also more frequently present in males than in females (*p* = 0.0070). However, the study only performed a univariate analysis without controlling for other factors. Another study by Li et al. identified regulators of 19 m^6^A RNA modifications in lung cancer as a predictive tool for personalized medicine via univariate Chi-square test using TCGA data from 1013 lung cancer patients (511 lung adenocarcinoma and 502 lung squamous carcinoma) and 109 controls [42]. Lung cancer samples were grouped according to hierarchical agglomerative consensus clustering. Although it was reported that clusters were related to sex (*p* < 0.001), the study did not look at overall survival in the two sample clusters for men and women separately. Overall, even though most studies reported a sex effect towards males, it is unclear if these epigenetic differences are due to sex biology or due to gendered differences in smoking behavior—a nuance which was not explored in these studies.

### 3.5. Synthesis of Results

The aim of this review was to assess the state of sex and gender reporting and analysis in cancer epigenetics research from 2010 onwards. Overall, the findings confirmed our hypothesis that there are variable standards in reporting and analyzing sex/gender within the cancer epigenetics field. Very few studies have explicitly provided a working definition of sex/gender variables or offered a robust analysis of sex/gender data. The conceptual conflations seen in the analysis of sex and gender-based variables can compromise the applicability and integrity of future meta-analyses or aggregate measures of epigenetic data.

## 4. Discussion

### 4.1. Summary of Evidence

After reviewing and appraising 111 total sources across the top 5 most prevalent cancers in the epigenetics field, only 17 studies (15.3%) explicitly stated sex and gender analysis to be their primary aim. Regardless, 103 studies (92.8%) performed a detailed sex or gender analysis, while the remaining 8 studies (7.2%) only stratified results by sex/gender. Notably, although sex/gender was promoted as a key facet in all eligible studies, only 7 studies (6.3%) provided an explicit well-defined and working definition of sex or gender, while the remaining 104 studies (93.7%) used the words “sex” or “gender” without providing a definition. Eighty-four studies (75.7%) conflated the concepts of sex and gender in its use (in comparison to WHO standards), while 44 studies (39.6%) were inconsistent with their usage of the terms “sex” and “gender” in the text. For the top five cancers, no studies included considerations of LGBTQIA+ or sex and gender minorities in any part of the text. In terms of the sex effect itself as reported by each study, 25 studies (22.1%) found an epigenetic effect in males, 26 studies (23.0%) found an effect in females, 47 studies (41.6%) found no differing effect between males and females, while 15 studies (13.3%) did not report any significant sex-related effect.

While real world global cancer trends indicated a higher prevalence for men than women at older ages for the top five cancers studied, most of the cancer studies found no sex-related epigenetic effect or did not report any effect altogether. Hepatocellular carcinoma and lung cancer had a greater number of studies which reported a male effect versus female effect, while head and neck cancers predominantly reported a female effect, with only one study reporting a male effect. Interestingly, analyses which disaggregated results by sex were least likely to occur among the head and neck literature review—a surprising find as head and neck cancers have the starkest sex bias of all the cancers under consideration [7]. While it cannot be ruled out whether sex or gender-based cancer effects occur at various biological levels (genetic, physiological, etc.), we did not investigate whether sex-based effects could be clearly discerned with statistical significance at the epigenetic level for the included studies. This does not mean that sex/gender effects do not exist. Real-world differences in sex-based prevalence could be mediated by mechanisms other than epigenetic ones. Instead, our study highlighted key issues in the definition, analysis, and communication of sex/gender data which can ultimately compromise the utility of sex and gender-based analysis in scientific studies.

For example, we observed great variability around the world over the usage, consistency, and meaning of sex and gender terms such as “male”, “female”, “men”, and “women”. It is important for these sex/gender concepts to be harmonized in conception and usage. From a scientific perspective, it fosters uniformity and rigor in the analysis of sex/gender as a biological variable, especially in aggregate measures such as EWAS or meta-analyses. From an ethical and social perspective, proper representation of these populations signify inclusion and awareness of the sex/gender differences occurring at the environmental or social level when conducting sex or gender-based analyses. Acknowledging non-binary sex/gender diversity, mentioning gender effect apart from sex effect in discussions, or incorporating gender-based differences into statistical analyses was also notably missing in most studies. Moving forward, filling in these knowledge gaps would ensure that scientific findings are representative of sex and gender factors that influence social and biological differences in the real world.

### 4.2. Limitations

There were some limitations that affected the quality of our scoping review. First, as there were no established systematic guidelines in place for the quality assessment of sex/gender data, we had to adapt our own protocol based on probing questions from the SAGER guidelines. Secondly, we recognize that different countries share different conceptions and connotations of the terms sex, gender, male, female, man, and woman. Although the international WHO definitions of sex and gender were used for the sake of uniform comparison in our study, we recognize that variable uses of these terms can exist without compromising the integrity of its underlying data analysis. However, this variability may prevent large-scale analyses or verification of study data by external researchers—which can ultimately compromise the applicability of inferences drawn from existing research.

### 4.3. Future Directions

Ultimately, the goal of this study was to capture differences and limitations in sex/gender reporting and data analysis across the modern cancer epigenetics field. Due to the breadth and heterogeneity of study types, a systematic review with meta-analysis was not conducted. As a result, it could not be confirmed if there were true statistically significant sex/gender effects at the epigenetic level, as studies within a single cancer type examined different aspects of the epigenome. Nonetheless, it remains a pertinent research question for future reviews of homogeneous studies. Future steps also include a detailed sex/gender analysis of all cancer types, rather than just the top five cancers, extracted from the initial cohort.

## 5. Conclusions

This review has undertaken a timely analysis of the state of sex and gender reporting of the top five cancer types in the global epigenetics field since 2010, including a quality assessment of the sex/gender data itself. Although many studies identified sex or gender differences to be a relevant variable in their cancer epigenetics investigation, very few studies have explicitly provided a working definition of sex/gender variables or offered a robust analysis of sex/gender data according to SAGER guidelines (outlined in Supplementary Materials S3). Little has been done to consolidate the use and analysis of sex/gender data in a uniform manner, sensitive to existing sex/gender-based imbalances in society. The conceptual conflations evident in the analysis of sex and gender-based variables can compromise the applicability and integrity of future meta-analyses or aggregate measures of epigenetic data.

We call for clear and directed guidelines regarding the use of sex/gender as a variable in epigenetics research. The SAGER guidelines and country-specific guidelines such as the U.S. National Institutes of Health (NIH) Policy on Sex as a Biological Variable are good starting points; however, they serve as a preliminary step for providing a broad overview of *reporting* sex and gender in research [43]. A more detailed guideline is needed, one which offers specific instructions on how to *discuss and analyze* sex and gender data in an equitable and inclusive way, such as requiring gender considerations in discussion sections, or including intersex as a variable in sex analysis. Guidelines should also explain how to incorporate sex/gender variables in the design and execution of their studies—in greater detail than current directives. For example, guidelines for sex/gender analyses should distinguish between studying a condition with substantial differences in incidence, severity, or progression, versus studying pathways or mechanisms (e.g., epigenetics) that are already known to exhibit sex/gender differences. Researchers should also include discussions on the influence of epidemiology, biology, or psychological behaviors related to the condition of interest—and whether the study is adequately designed to examine such factors.

Within the broader research community, more incentives are needed, from journal editors and fundings agencies alike, to harmonize usage of sex/gender terminology and hold stricter standards regarding the implementation of the SAGER guidelines in epigenetics research. As a starting point, we propose several recommendations to improve sex and gender-based analysis in cancer epigenetics research (Box 2).

Box 2 **Recommendations for the Improvement of Sex and Gender-Based Analysis.**

❖ Define a clear conceptual framework for the sex/gender terms used and list the variables for each sex/gender category (e.g., male, female, intersex) in the study.
❖ Include a variety of sex or gender-based covariates in the methodological analysis or discussion sections of the study, with considerations of sex and gender minorities treated distinctively.
  ➢ For example, gender could be incorporated into statistical analysis by controlling for lifestyle factors (diet, access to screening and diagnosis, smoking habits).
❖ Ensure that all researchers are trained in sex and gender-based analysis methods to create awareness of an-alytical and reporting bias.
❖ Inquire and motivate international scientific organizations to delineate standards for the usage and inter-pretation of sex/gender data in the epigenetics and genetics field.
❖ For authors and journals, ensure higher standards of reporting and publishing sex and gender-based data.

Overall, we hope this study will encourage future actionable steps to improve the rigor of sex/gender-based analysis in the cancer epigenetics field, and health research as a whole.

## Figures and Tables

**Table 1:** Summary of Sex/Gender Differences Stratified by Cancer Type.

Cancer Type	Findings
Colorectal Cancer	32 total studies The majority (18 studies, 56.3%) did not report significant sex or gender-based epigenetic differences. 9 studies (28.1%) reported a sex-based effect in females. 5 studies (15.6%) reported a sex-based effect in males.
Gastric Cancer	16 total studies The majority (10 studies, 62.5%) either did not report or reported no significant sex/gender differences. 2 studies (12.5%) reported an epigenetic effect in females. 3 studies (18.75%) reported an epigenetic effect in males.
Head and Neck Cancers	15 total studies The majority (9 studies, 60%) did not find a sex/gender effect (5 studies reported no effect, 4 studies did not report any effect). 5 studies (33.3%) reported an epigenetic effect in females. 1 study (0.07%) reported an epigenetic effect in males.
Hepatocellular Carcinoma	22 total studies The majority (8 studies, 36.4%) reported no significant sex or gender-based epigenetic differences. 2 studies (9.1%) did not report any specific sex effect. An equal majority (8 studies, 36.4%) reported an epigenetic effect in males. 4 studies (18.2%) reported an epigenetic effect in females.
Lung Cancer	26 total studies 7 studies (26.9%) reported no significant sex related effect. 7 studies (26.9%) did not report any sex/gender effects. A slight majority (8 studies, 30.5%) reported an epigenetic-based effect in males. 5 studies (19.2%) reported an effect in females.

## Data Availability

Besides the data included in the full text and supplemental materials, any additional datasets generated during and/or analyzed during the current study are available from the corresponding author on reasonable request.

## Supplementary Materials

The following supporting information can be downloaded at: https://www.mdpi.com/article/10.3390/cancers15174207/s1, Material S1: Summary of Search Strategy, Material S2: Detailed PRISMA-ScR Methods, Material S3: SAGER Guidelines—Sex & Gender Quality Appraisal Checklist, Material S4: Critical Appraisal Results.
