# Supplementary material for: Studies in Cancer Epigenetics through a Sex and Gendered Lens: A Comprehensive Scoping Review
Source: Cancers (Basel). 2023 Aug 22;15(17):4207. doi: 10.3390/cancers15174207 (PMC10486657; doi:10.3390/cancers15174207)
Supplement: Supplementary file 1 [file cancers-15-04207-s001.zip › Material S1.pdf]

S1: Summary of Search Strategy

Database	Search Terms	Date Searched	Number of Hits
Google Scholar	"cancer" drugs and "~epigenetics" and intext:sex and gender	Aug 12, 2021	first 219 results collected out of 17,600
Scopus	(TITLE-ABS-KEY (cancer AND drugs) AND TITLE-ABS-KEY (epigenetics) AND TITLE-ABS-KEY (sex) OR TITLE-ABS-KEY (gender)) AND (LIMIT-TO (PUBYEAR , 2021) OR LIMIT-TO (PUBYEAR , 2020) OR LIMIT-TO (PUBYEAR , 2019) OR LIMIT-TO (PUBYEAR , 2018) OR LIMIT-TO (PUBYEAR , 2017) OR LIMIT-TO (PUBYEAR , 2016) OR LIMIT-TO (PUBYEAR , 2015) OR LIMIT-TO (PUBYEAR , 2014) OR LIMIT-TO (PUBYEAR , 2013) OR LIMIT-TO (PUBYEAR , 2012) OR LIMIT-TO (PUBYEAR , 2011) OR LIMIT-TO (PUBYEAR , 2010)) AND (EXCLUDE (DOCTYPE , "ch") OR EXCLUDE (DOCTYPE , "ed") OR EXCLUDE (DOCTYPE , "no") OR EXCLUDE (DOCTYPE , "cp") OR EXCLUDE (DOCTYPE , "le") OR EXCLUDE (DOCTYPE , "sh")) AND (EXCLUDE (LANGUAGE , "Chinese") OR EXCLUDE (LANGUAGE , "Croatian") OR EXCLUDE (LANGUAGE , "French") OR EXCLUDE (LANGUAGE , "Polish") OR EXCLUDE (LANGUAGE , "Russian")) AND (EXCLUDE (SRCTYPE , "k"))	Aug 12, 2021	194
PubMed	("Epigenomics"[MeSH Terms] OR "epigen*"[All Fields]) AND (("Neoplasms"[MeSH Terms] OR "Antineoplastic Agents"[Mesh] OR "Antineoplastic Agents" [Pharmacological Action] OR "cancer*"[all] OR adenocarcinoma*[tiab] OR carcinoma*[tiab] OR tumor*[tiab] OR tumour*[tiab] OR chemotherap*[tw] OR antitumo*[tw] OR anticancer*[tw] OR antineoplastic[tw]) NOT ("Leukemia"[ti] OR "leukaemia*"[ti] OR "hematolog*"[ti] OR haematolog*[ti] OR "Lymphoma*"[ti] OR "Multiple Myeloma"[ti] OR "Breast"[TI] OR "Cervi*"[TI] OR "Ovar*"[TI] OR "Uterine"[TI] OR endometri*[ti] OR "Prostate"[TI] OR "Testicular"[TI])) AND ("Sex"[MeSH Terms] OR "Gender Identity"[MeSH Terms] OR "Sex"[tiab] OR "sexual*"[tiab] OR "gender*"[tiab]) AND ("Antineoplastic Agents"[Mesh] OR "Antineoplastic Agents" [Pharmacological Action] OR therap*[tw] OR drug*[tw] OR treatment*[tw] OR medication*[tw] OR radiation[tw] OR chemotherap*[tw] OR antitumo*[tw] OR anticancer*[tw] OR antineoplastic[tw]) NOT (("animals"[MeSH Terms] NOT "humans"[MeSH Terms]) OR "mice"[ti]) AND "2011/07/21 00:00":"3000/01/01 05:00"[Date - Publication]	August 17, 2021	689

PubMed (cancer drugs only)	("Epigenomics"[MeSH Terms] OR "epigen*"[All Fields]) AND (("Neoplasms"[MeSH Terms] OR "Antineoplastic Agents"[Mesh] OR "Antineoplastic Agents" [Pharmacological Action] OR "cancer*"[all] OR adenocarcinoma*[tiab] OR carcinoma*[tiab] OR tumor*[tiab] OR tumour*[tiab] OR chemotherap*[tw] OR antitumo*[tw] OR anticancer*[tw] OR antineoplastic[tw]) NOT ("Leukemia"[ti] OR "leukaemia*"[ti] OR "hematolog*"[ti] OR haematolog*[ti] OR "Lymphoma*"[ti] OR "Multiple Myeloma"[ti] OR "Breast"[TI] OR "Cervi*"[TI] OR "Ovar*"[TI] OR "Uterine"[TI] OR endometri*[ti] OR "Prostate"[TI] OR "Testicular"[TI])) AND ("Sex"[MeSH Terms] OR "Gender Identity"[MeSH Terms] OR "Sex"[tiab] OR "sexual*"[tiab] OR "gender*"[tiab]) AND ("Antineoplastic Agents"[Mesh] OR "Antineoplastic Agents" [Pharmacological Action] OR therap*[tw] OR drug*[tw] OR treatment*[tw] OR medication*[tw] OR radiation[tw] OR chemotherap*[tw] OR antitumo*[tw] OR anticancer*[tw] OR antineoplastic[tw]) NOT (("animals"[MeSH Terms] NOT "humans"[MeSH Terms]) OR "mice"[ti]) AND "2011/07/21 00:00":"3000/01/01 05:00"[Date - Publication]	August 27, 2021	308
Total Hits			1410

Exclusion criteria (for first and second round screening):

- the study was not about cancer research
- the study was not about epigenetics research
- the study did not provide a detailed or specific discussion or analysis of sex and/or gender
- the study mentioned cancer/epigenetics/sex/gender as a word but did not discuss the topic in detail
- the study did not involve human bodies or human-derived samples (i.e. animal studies)
- articles did not have a full-text available
- articles were conference reports or pre-prints
- articles were not in English
