# Supplementary material for: Studies in Cancer Epigenetics through a Sex and Gendered Lens: A Comprehensive Scoping Review
Source: Cancers (Basel). 2023 Aug 22;15(17):4207. doi: 10.3390/cancers15174207 (PMC10486657; doi:10.3390/cancers15174207)
Supplement: Supplementary file 1 [file cancers-15-04207-s001.zip › Material S2.pdf]

S2: Detailed PRISMA-ScR Methods

A scoping review was conducted to elucidate the trends in sex/gender analysis in cancer epigenetics research. The protocol was developed and reported following the PRISMA extension for scoping reviews (PRISMA-ScR)[1].

Protocol and Registration

The protocol was registered and made publicly available on Open Science Framework (OSF) on 2021-11-16 (DOI: 10.17605/OSF.IO/CH2DP).

Eligibility Criteria

Population

The subject of interest was humans only. The study was included if it involved the use of human biological samples in any capacity (e.g. human cell lines, tissue samples, human bodies in clinical or epidemiological settings). Studies conducted only in animals were excluded. Studies that used both animal and human subjects, but in which the primary clinical outcome was not conducted in humans, were also excluded.

Topics of Study

Peer-reviewed publications were included if it explicitly discussed or statistically analyzed all three topics in the full text: cancer, epigenetics, and sex/gender. Studies which did not mention all three topics were excluded.

'Cancer' as a topic was included if the study was about cancer cell lines, neoplasms, cancer epidemiology, specific or subtypes of cancers, or a review of existing cancer literature. It was excluded if 'cancer' appeared as a keyword in the text but there was no significant discussion on the topic, or if cancer was one of multiple topics listed, but it was not discussed within the context of cancer epigenetics.

'Epigenetics' as a topic was included if the study discussed DNA methylation, chromosome modification or structure, modulators of the epigenome, and regulatory elements (like miRNA or sncRNA) that have a direct regulatory impact on the epigenome, including X-chromosome inactivation. This included studies which reviewed epigenetic mechanisms and regulatory pathways. 'Epigenetics' as a topic was excluded if the study mentioned the 'epigenome', 'epigenetics' or any epigenetic elements as a keyword but did not elaborate on it, as well as study that mention factors which influence the epigenome (like sex hormones), but were not considered strictly to be part of the epigenome.

'Sex' and/or 'gender' as a topic was included if the topic of sex or gender was discussed using terminology such as sex, gender, male, female, masculine, feminine, intersex, transgender, non-binary, etc. For basic and clinical studies, the study was included if sex/gender was analyzed as a

biological variable. For example, the study analyzed if sex (as a biological variable) produced a differing effect on methylation state. The study was excluded if the word "sex" or "gender" was mentioned as a keyword in the text with no detailed discussion on the topic, or if keywords related to sex or gender (e.g. male, female, man, woman, intersex) could not be searched in the full text.

Time Frame

A pilot search was conducted to gauge the overall publication trends mentioning sex or gender in the cancer epigenetics field. As most articles appeared after 2010, only articles that were published from January 2010 - August 2021 were included.

Forms of Publication

All forms of academic, peer-reviewed, journal publications were included, so long as they met the topic requirements. Gray literature that was not peer-reviewed was excluded.

Language

Any publication which was not in English or had an English translation was excluded.

Information Sources

Three databases were used: PubMed, Google Scholar and Scopus.

Search Strategy

The search was designed in consultation with an experienced health sciences librarian (G.G.) and conducted by K.H. in August 2021. The keywords used were epigenetics, cancer, sex, and gender, with MeSH terms for each keyword used when available. The search using the same keywords and time period was repeated on each of the three search engines: PubMed, Google Scholar and Scopus. For Google Scholar, only the first 210 results out of 17,500 hits were collected for conciseness. The search strategies with exact parameters used for each database are listed in Appendix A. A total of 1411 hits were extracted from all 3 databases and 4 search results.

Selection of Sources

Data Management

Abstracts and bibliographic information were collected and exported to Zotero and Excel. Inclusion/exclusion decisions and removal of duplicates were managed on Excel. All analyses and data extraction were conducted on Excel.

Data Charting Process

After the removal of duplicates and retracted articles (n = 390), 1020 hits remained. Two reviewers K.H. and N.P. independently performed an initial screening of the remaining results by abstract and title based upon the exclusion criteria outlined in Appendix A, for a total of 428 hits (n = 592 excluded). Discrepancies were resolved via consensus. Two reviewers N.P and A.B. independently

performed a second screening of the remaining results through reading the full-text in detail (records excluded n = 243). Discrepancies were resolved via consensus or third reviewer K.H., as necessary, resulting in 185 documents. The studies were then classified by cancer type and for conciseness, only the studies from the top 5 cancer types with the most number of studies were included, for a remaining total of 111 studies. The final selection of studies included for data analysis was recorded, and the reason for exclusion was annotated in Excel.

Data Collection

The data items of interest were extracted manually and recorded on Excel, by K.H, S.S.J., and R.L. The full texts of the remaining 111 hits were read and data items were extracted by two independent reviewers at all times.

Data Items

The following data items were extracted: title, author, year of publication, country of origin (based upon first authorship and/or funding country), study type, cancer type, aim of study, characteristics of the sample population (e.g. sample size), characteristics of the control population, if the primary aim of the study was to examine sex/gender, if any definition of sex/gender was provided, type of statistical analysis used for sex/gender analysis, sex/gender characteristics of study population, sex/gender variables, sex/ gender findings, sex/gender effect, statistical significance of sex/gender findings, and inclusion of non-binary sex/gender populations. A comparative data extraction against WHO guidelines was used to determine if there was a conceptual conflation of sex and gender, if the terms "sex" and "gender" were used interchangeably, and an assessment of gender discourse (according to the Gender-Responsive Assessment Scale) for studies that included gender analysis.

For the 'study type category, each hit was codified into additional subcategories:

- Basic research studies on Y-linked genes
- Basic research studies on methylation
- Basic research studies on histone modification
- Basic research studies on chromatin conformation
- Basic research studies on non-coding RNA (ncRNA)
- Basic research studies on drugs or drug interactions
- Clinical research involving case control studies
- Clinical research involving cohort studies
- Clinical research involving correlational studies
- Clinical research involving cross-sectional studies
- Systematic reviews
- Meta-analyses
- Non-systematic reviews (e.g. narrative review, scoping review)

Reviews were included in the study to capture more extensive sex/gender discussions, with reviews often providing valuable commentary on gender in particular.

For the ‘cancer type’ category, each study was coded into additional subcategories based on the main topic of focus in the study: biliary tract, brain, breast, colorectal, esophageal, gastric, general or non-specific cancers, germ cell tumor, hematological, head and neck, hepatocellular carcinoma, lung, melanoma, meningioma, non-melanoma skin, pancreatic, renal, sarcoma, thyroid, and urothelial. Of this list, only the top 5 cancers (colorectal, gastric, head and neck, hepatocellular carcinoma, and lung; representing a total of 111/185 studies) were chosen for data analysis.

The following table lists the framework applied for the remaining codified data items.

Table S1: Legend for Data Extraction Items

Data Item	Variable	Definitions, Assumptions, or Simplifications
**Is the primary aim to study sex or gender?**	Yes	The main goal of the study is to examine sex/gender differences in cancer epigenetics.
	No	The aim of the study is about some other aspect of cancer epigenetics, and/or sex/gender was included without explicit emphasis.
**Is sex/gender defined?**	Yes	A definition for sex or gender is provided explicitly in the text.
	No	A definition is not provided.
**Is sex/gender analyzed in depth?**	Yes	A significant portion of the study utilizes some qualitative discussion or quantitative/statistical analysis of sex/gender, with the methods of SGBA being well-described.
	No	The study may report sex/gender characteristics, but does not describe how sex/gender is analyzed in relation to the study’s aims.
**Is there inclusion of non-binary sex/gender minorities?**	Yes	The study mentions or accounts for non-binary sex/gender populations like transgender, intersex, two-spirit, etc.
	No	The study only categorizes or discusses sex/gender as male/female or man/woman in the binary.
**Is there conflation of sex and gender concepts?**	Yes	The context, discourse or cognizant understanding of the term sex or gender does not follow the respective definition provided by the WHO.
	No	The use of the term sex or gender follows the respective definition provided by the WHO.
**Are sex/gender terms used interchangeably?**	Yes	The terms sex and gender are used interchangeably throughout the study to refer to the same concept.
	No	The terms sex and gender are used separately and consistently.

	Only once	The terms sex and gender are used interchangeably only once in the study.
	Unclear	It cannot be determined based on context if the author was mistakenly referring to sex as gender or vice versa.
**Overall sex/gender effect**	Effect within males	The study has detected a significant positive or negative effect or trend within males for the epigenetic variable of interest.
	Effect within females	The study has detected a significant positive or negative effect or trend within females for the epigenetic variable of interest.
	No effect	The study did not find any significant positive or negative sex or gender-based effect for either males or females.
	Not reported	Sex/gender effect was not reported for the variable of interest.
**Gender-Responsive Assessment Scale according to WHO guidelines**	Gender unequal	The study discusses gender in a way that perpetuates gender inequality by privileging men over women, or vice versa
	Gender blind	The study discusses gender in a way that ignores gender norms, roles and relations based on the principle of being fair to everyone
	Gender sensitive	The study discusses gender in a way that considers gender roles, relations and norms, but provides no remedial action
	Gender specific	The study discusses gender in a way that considers gender roles, relations and norms, by targeting men or women explicitly
	Gender transformative	The study discusses gender in a way that addresses the causes of gender-based health inequities and includes ways to transform harmful gender norms, relations and roles

Critical Appraisal of Individual Sources of Evidence

There currently lacks a universally recognized and rigorous tool for the critical appraisal (quality assessment) of sex and gender-based analysis in basic and clinical research. Instead, the critical appraisal scheme for our study was adapted from the internationally recognized SAGER Guidelines – Sex & Gender Quality Appraisal Checklist[4]. They were originally developed to offer a comprehensive outline for reporting of sex and gender information in study design, data analysis, and interpretations of findings. These guidelines were repurposed to assess the state of sex and gender reporting, scope of use, and rigor in quantitative analysis in each study (excluding review articles and one study that only looked at males). Two independent screeners (S.S.J. and R.L.) performed the quality appraisal, with discrepancies resolved via consensus. An agreement was reached regarding the interpretation of each of the questions derived from the checklist and the study elements required to satisfy each of the methodological conditions. An overview of the questions and data coding process is provided in Appendix C. Both reviewers noted whether or not a study met the various aspects of the SAGER guidelines and results were recorded on Excel. Any discrepancies were resolved by discussion between S.S.J. and R.L. and consensus was

mediated by K.H. when necessary. The critical appraisal was used to report if any sex or gender biases existed for each field or type of cancer research. The results of the appraisal were used in conjunction with the results of the data extraction to provide a comprehensive report on the state of sex/gender findings in the cancer epigenetics field.
