# Supplementary material for: Studies in Cancer Epigenetics through a Sex and Gendered Lens: A Comprehensive Scoping Review
Source: Cancers (Basel). 2023 Aug 22;15(17):4207. doi: 10.3390/cancers15174207 (PMC10486657; doi:10.3390/cancers15174207)
Supplement: Supplementary file 1 [file cancers-15-04207-s001.zip › Material S3.pdf]

S3: SAGER Guidelines – Sex & Gender Quality Appraisal Checklist

Adapted from Table 2 (Appendix) - Authors' checklist for gender-sensitive reporting

Heidari, S., Babor, T. F., De Castro, P., Tort, S. & Curno, M. Sex and Gender Equity in Research: rationale for the SAGER guidelines and recommended use. *Res Integr Peer Rev* **1**, 2 (2016).

Research Approaches

Are sex and/or gender relevant to the topic of the study?

- Possible responses: *Yes, No, Unclear*
- Comments: *add any details from the study relevant to the question*
    - *E.g. "sex was a relevant topic to the study because the study was about the methylation patterns on the X chromosome"*

Are the concepts of gender and/or sex used in the study?

- Possible responses: *Yes, No, Unclear*
- Comments: *add any details from the study relevant to the question*

Have the concepts of gender and/or sex been explicitly defined?

- Possible responses: *Yes, No, Unclear*
- Comments: *add any details from the study relevant to the question*

Is it clear what aspects of gender and/or sex are being examined in the study?

- Possible responses: *Yes, No, Unclear*
- Comments: *add any details from the study relevant to the question*

Given existing knowledge in the relevant literature, are there plausible gender and/or sex factors that should have been considered?

- Possible responses: *Yes, No, Unclear*
- Comments: *add any details from the study relevant to the question*

Has consideration of sex/gender (or lack thereof) been described in the design of the study?

- Possible responses: *Yes, No, Unclear*
- Comments: *add any details from the study relevant to the question*

Research Questions and Hypotheses

Does the research question(s) or hypothesis/aim make reference to gender and/or sex, or relevant groups or phenomena? (e.g., differences between males and females, differences among women, seeking to understand a gendered phenomenon such as masculinity)

- Possible responses: *Yes, No, Unclear*
- Comments: *add any details from the study relevant to the question*

Literature Review or Introduction

Does the literature review or introduction cite prior studies that support the existence (or lack) of significant differences between women and men, boys and girls, or males and females?

- Possible responses: *Yes, No, Unclear*
- Comments: *add any details from the study relevant to the question*

Does the literature review or introduction point to the extent to which past research has taken gender or sex into account?

- Possible responses: *Yes, No, Unclear*
- Comments: *add any details from the study relevant to the question*

Research Methods

Is the sample population appropriate to capture gender and/or sex-based factors?

- Possible responses: *Yes, No, Unclear*
- Comments: *add any details from the study relevant to the question*

Is it possible to collect data that are disaggregated by sex and/or gender?

- Possible responses: *Yes, No, Unclear*
- Comments: *add any details from the study relevant to the question*

Are the inclusion and exclusion criteria well justified with respect to sex and/or gender?

- Possible responses: *Yes, No, Unclear*
- Comments: *add any details from the study relevant to the question*

Is the data collection method proposed in the study appropriate for investigation of sex and/or gender?

- Possible responses: *Yes, No, Unclear*
- Comments: *add any details from the study relevant to the question*

Is the analytic approach appropriate and rigorous enough to capture gender and/or sex-based factors?

- Possible responses: *Yes, No, Unclear*
- Comments: *add any details from the study relevant to the question*

Discussion/Limitation

Have all data been reported and disaggregated by sex and/or gender?

- Possible responses: *Yes, No, Unclear*
- Comments: *add any details from the study relevant to the question*

Has sex and gender-based analysis (or lack thereof) been mentioned and discussed in the discussion and limitations sections?

- Possible responses: *Yes, No, Unclear*
- Comments: *add any details from the study relevant to the question*

Ethics

Does the study design account for the relevant ethical issues that might have particular significance with respect to gender and/or sex? (e.g., inclusion of pregnant women in clinical trials)

- Possible responses: *Yes, No, Unclear*
- Comments: *add any details from the study relevant to the question*
