# Supplementary material for: Studies in Cancer Epigenetics through a Sex and Gendered Lens: A Comprehensive Scoping Review
Source: Cancers (Basel). 2023 Aug 22;15(17):4207. doi: 10.3390/cancers15174207 (PMC10486657; doi:10.3390/cancers15174207)
Supplement: Supplementary file 1 [file cancers-15-04207-s001.zip › Material S4.pdf]

S4: Critical Appraisal Results

SAGER Guideline Questions	# of 'Yes' Responses	# of 'No' Responses	# of 'Unclear' Responses	Comments
Are sex and/or gender relevant to the topic of the study?	101	0	0	
Are the concepts of gender and/or sex used in the study?	101	0	0	
Have the concepts of gender and/or sex been explicitly defined?	100	1	0	If no, authors used the term gender when they mean sex or used the terms sex and gender interchangeably.
Is it clear what aspects of gender and/or sex are being examined in the study?	94	7	0	If no, aspects of sex (e.g. chromosomes, sex hormones, etc.) or gender (e.g. expression, social roles) were not specified.
Given existing knowledge in the relevant literature, are there plausible gender and/or sex factors that should have been considered?	99	2	0	If no, authors considered appropriate sex factors
Has consideration of sex/gender (or lack thereof) been described in the design of the study?	17	84	0	If no, subgroup analyses by sex were not described in the design.
Does the research question(s) or hypothesis/aim make reference to gender and/or sex, or relevant groups or phenomena? (e.g., differences between males and females, differences among	8	93	0	If no, the research question or hypothesis/aim did not consider sex and/or gender.

women, seeking to understand a gendered phenomenon such as masculinity)				
Does the literature review or introduction cite prior studies that support the existence (or lack) of significant differences between women and men, boys and girls, or males and females?	8	93	0	If no, the introduction did cite prior studies that support or the existence or lack of sex and/or gender differences.
Does the literature review or introduction section indicate the extent to which past research has taken gender or sex into account?	7	94	0	If no, the introduction did not mention the extent to which previous research investigated sex and/or gender.
Is the sample population appropriate to capture gender and/or sex-based factors?	101	0	0	
Is it possible to collect data that are disaggregated by sex and/or gender?	101	0	0	
Are the inclusion and exclusion criteria well justified with respect to sex and/or gender?	0	0	101	If unclear, inclusion and exclusion criteria did not explicitly address sex and/or gender, but males and females were enrolled in the study.
Is the data collection method proposed in the study appropriate for investigation of sex and/or gender?	7	94	0	If no, authors conflated the terms sex and gender.

Is the analytic approach appropriate and rigorous enough to capture gender and/or sex-based factors?	11	90	0	If no, authors did not perform sex and/or gender analyses, performed univariate analyses for sex without adjusting for other clinicopathological factors or did not perform subgroup analyses by sex.
Have all data been reported and disaggregated by sex and/or gender?	13	87	0	If no, authors did not report and disaggregate data by sex and/or gender.
Has sex and gender-based analysis (or lack thereof) been mentioned and discussed in the discussion and limitations sections?	41	60	0	If no, authors did mention sex and/or gender based analysis in the discussion and limitations section.
Does the study design account for the relevant ethical issues that might have particular significance with respect to gender and/or sex? (e.g., inclusion of pregnant women in clinical trials)	13	88	0	If no, promising findings for one sex could be lost due to failure to disaggregate results.
